# Expression of the prostaglandin F synthase AKR1B1 and the prostaglandin transporter SLCO2A1 in human fetal membranes in relation to spontaneous term and preterm labor

**DOI:** 10.3389/fphys.2014.00272

**Published:** 2014-07-30

**Authors:** Hana A. Alzamil, Joya Pawade, Michel A. Fortier, A. López Bernal

**Affiliations:** ^1^Department of Physiology, King Saud UniversityRiyadh, Saudi Arabia; ^2^Pathology, University Hospitals Bristol Haemato-Oncology Diagnostic Service, Bristol Royal InfirmaryBristol, UK; ^3^Axe Reproduction, Santé Périnatale et Pédiatrie, Centre Hospitalier Universitaire de Québec, Université LavalQC, Canada; ^4^Academic Unit of Obstetrics and Gynaecology, School of Clinical Sciences, University of BristolBristol, UK

**Keywords:** prostaglandins, cytokines, placenta, parturition, preterm birth, uterus

## Abstract

**Background**: Human labor is a complex series of cellular and molecular events that occur at the materno-fetal and uterine levels. Many hypotheses have been proposed for the initiation of human labor, one hypothesis suggests that maturation of the fetus releases a signal in the amniotic fluid that will be transmitted to myometrium via the fetal membranes and initiate uterine contractions. There is strong evidence that prostaglandins (PGs) play a central role in initiation and progression of human labor.

**Objectives**: In this study we intended to investigate the expression of prostaglandin F synthase and the prostaglandin transporter in the human fetal membranes and to explore the relationship between cytokines and PGs in the mechanism of human labor.

**Methods**: We used fetal membranes obtained before labor at term and after spontaneous labor at term or preterm to identify the changes in prostaglandin F synthase (AKR1B1) and human prostaglandin transporter (SLCO2A1) proteins in relation to parturition. Using fetal membranes explants we tested the effect of cytokines (interleukin-1 and tumor necrosis factor alpha) on PG production and the concomitant changes in cyclooxygenase-2 (PTGS2), AKR1B1 and SLCO2A1 expression.

**Results**: Expression of PTGS2 and AKR1B1 was upregulated in the fetal membranes in association with term labor while SLCO2A1 was downregulated with advancing gestation and during term labor. Before labor, IL-1 increased the expression of PTGS2, however during labor TNF upregulated PTGS2 and AKR1B1 proteins.

**Conclusions**: The prostaglandin F synthase AKR1B1 is upregulated while prostaglandin transporter is downregulated during term labor. The amnion is more responsive than choriodecidua to stimulation with pro-inflammatory cytokines. The mechanisms of term and preterm labor are different.

## Introduction

Labor is the result of strong uterine contractions that lead to expulsion of the fetus to the extrauterine environment. The incidence of preterm births is increasing and represents one of the most challenging clinical problems all over the world. In the United States the preterm birth rate increased from 9.5% in 1981 to 12.7% in 2005; the reported rates of preterm birth in other developed countries and Europe range from 5 to 9% (Goldenberg et al., 2008). At St Michael's Hospital in Bristol the incidence of preterm births is approximately 8% of all births (Yuan et al., 2010).

The trigger of the onset of human labor is still unknown, despite the impressive gains in clinical, physiological, and biochemical knowledge. Some studies suggest that there are several mechanisms for the onset of labor in women, such as paracrine/autocrine events, fetal hormonal changes and overlapping maternal/fetal factors (Weiss, 2000). Interaction between several pathways might be essential to initiate the process of labor and may include changes in estrogen and progesterone levels, elevation of corticotropin-releasing hormone (CRH), increased production of prostaglandins (PGs) and increased sensitivity of the uterus to oxytocin (Navitsky et al., 2000). However, it is not known whether labor is due to stimulation of uterine contraction or loss of inhibitory mechanisms that keep the uterus quiescent during pregnancy (Lopez Bernal et al., 1995).

There is strong evidence that PGs play a central role in initiation and progression of human labor. Generally it is believed that the main sources for PGs found in the amniotic fluid are the fetal membranes and decidua (Gibb, 1998; Olson and Ammann, 2007). The maternal plasma level of PGF metabolites increases with the onset of labor at term as well as preterm (Sellers et al., 1981). Locally produced PGs can stimulate uterine contractions by: (1) direct effect via their own receptors in the myometrium (Senior et al., 1993), (2) increasing the sensitivity of the myometrium to oxytocin through upregulation of oxytocin receptors (OTR) (Chan and Chen, 1992), (3) upregulation of myometrial gap junctions (Ivanisevic et al., 2001). Oxytocin and PGs have a positive interaction which might be vital for coordinated and efficient uterine contraction at the time of labor (Arias, 2000).

The PGs biosynthesis cascade involves different steps. First, arachidonic acid (AA) is liberated from membrane phospholipids principally through cytosolic phospholipase A_2_. AA is then converted into prostaglandin H_2_ (PGH_2_), a relatively unstable compound, by the action of prostaglandin H synthases (PTGS) also known as cyclooxygenase enzymes and then into bioactive PGs by terminal synthases such as TXA-, PGE-, and PGF-synthases. PTGS enzymes have received most of the attention in the literature; many studies have investigated their changes with labor and the effect of blocking their actions in the management of preterm labor (Fuentes et al., 1996; Slater et al., 1999; Johnson et al., 2002; Stika et al., 2002). Terminal synthase pathways beyond PTGS, other than PGE synthases, have not been well-investigated, although they might be excellent specific target in the prevention of preterm labor. There are three different pathways for the synthesis of the uterotonic PGF_2α_: (1) from PGH_2_ by PGH 9-, 11-endoperoxide reductase, (2) from PGD_2_ by PGD 11-ketoreductase, (3) from PGE_2_ by PGE 9-ketoreductase (Watanabe, 2002).

The main source of PGF_2α_ is the direct conversion of PGH_2_ by PGH_2_ 9, 11-endoperoxide reductase; this activity is known as PGF synthase (Watanabe et al., 1985). Only small amounts of PGF_2α_ are produced through reduction of PGE_2_ by carbonyl reductase 1. AKR1B1 has recently been identified as a functional PGF synthase in the human uterus (Bresson et al., 2011) and based on its molecular weight, amino acid sequence and substrate specificity it has been assigned to the aldo-keto reductase family (Suzuki et al., 1999). Although PGF_2α_ is known to increase at the time of labor and proved to induce myometrial contractions, to date there is no information about PGF synthase expression and regulation in human fetal membranes during pregnancy and labor (Helliwell et al., 2004).

PGs are of lipid nature; however they diffuse poorly through plasma membranes because they exist as charged species at physiological pH (Schuster, 1998). The mechanism of PG transport across the plasma membrane is not well-known, however different models have been proposed such as simple diffusion, countercurrent transfer and carrier-mediated transport (Banu et al., 2005). A prostaglandin transporter (SLCO2A1) is broadly distributed in human tissues and it is likely that it has a role in clearance and metabolism of endogenous prostanoids (Lu et al., 1996). The functional uptake-carrier SLCO2A1 is a 12-transmembrane protein which has high affinity for PGE_2, PGF_2α_, and PGD_2_. During the menstrual cycle the expression of SLCO2A1 in human endometrium was found to be modulated at mRNA and protein levels, (Kang et al., 2005). To date there is very little information regarding the distribution of prostaglandin F synthase (AKR1B1) and prostaglandin transporter (SLCO2A1) in the human fetal membranes in relation to term and preterm labor. AKR1B1 and SLCO2A1 are more abundant in choriodecidua than in the amnion (Breuiller-Fouche et al., 2010; Phillips et al., 2011).

Cytokines provide a major soluble intracellular signaling network which allows biochemical cross-talk between the fetus and the uterus. Moreover it has been proposed that regulation of prostaglandin production by pro-inflammatory cytokines is an important aspect of the mechanism of labor (Hansen et al., 1999). Paracrine and autocrine interactions between pro-inflammatory cytokines, especially interleukin-1 (IL-1) and tumor necrosis factor-alpha (TNF), and PGs have been demonstrated in term human decidua (Norwitz et al., 1992; Hansen et al., 1999). There is strong evidence that fetal membranes are involved in the process of human labor via release of PGs, however there are relatively few studies comparing PG production from membranes obtained before labor and those obtained after term and preterm labor.

The purpose of this manuscript was to examine the distribution of key enzymes for PG synthesis and transport in the fetal membranes/decidua interface and to look for differences in expression in relation to gestational age and the absence or presence of term and preterm labor. Moreover we intended to test the effect of IL-1 and TNF on PG release, using an *ex-vivo* fetal membranes model that preserves the anatomical integrity of the tissues. We have conducted detailed experiments on the expression of terminal PGF synthase and transporter in fetal membranes, and assessed the effect of pro-inflammatory cytokines on their protein expression levels. This study provides novel information on the source of triggers of human labor and the roles of inflammatory cytokines in the mechanism of parturition that will help in improving the management of preterm labor.

## Materials and methods

### Collection of tissues

The study was approved by the North Somerset and South Bristol Research Ethics Committee. Placentas were collected from delivery suite in St Michael's Hospital, Bristol, after women were informed of the study and signed a consent form. All women had singleton gestations and experienced spontaneous labor at term (37–42 weeks gestation) or preterm (25–36 weeks), or had an elective cesarean section before any signs of labor at term. Patients admitted for elective preterm deliveries, due to placenta praevia or preeclampsia, or induction of labor (IOL) at term were also recruited (Table 1). The following groups of women were included: Term not in labor (TNIL), preterm not in labor (PNIL), spontaneous preterm labor (SPL), spontaneous term labor (STL), and IOL. Patients with multiple pregnancies, prolonged rupture of the membranes, or any signs of infection, including histological chorioamnionitis, were excluded. All tissues were carried to the laboratory in sterile cold saline and used immediately for tissue culture, fixed for immunochemistry, or snap frozen and stored in liquid nitrogen till used.

**Table 1:** **Clinical details of patients whose samples were used in this study**.

**Mean (range)**	**TNIL**	**PNIL**	**SPL**	**STL**	**IOL**
Number (*n*)	7	9	8	9	9
Parity	1 (1–2)	0	1 (0–2)	1 (0–2)	0
Gestational age (weeks ^+days^)	39 (39–40)	33^+4^ (27–36)	32^+2^ (25–36)	40 (39–40)	41^+4^(39–42)
Duration of labor (h:min)	_	_	5:21 (1:8–17:7)	7:15 (00:20–11:8)	9:26 (6:7–12:44)
Fetal weight (gm)	3790 (3300–4000)	1719 (585–2930)	1674 (780–2100)	3438 (2790–4000)	3713 (2870–4380)

*TNIL, Term not in labor; PNIL, preterm not in labor; SPL, spontaneous preterm labor, STL, spontaneous term labor; and IOL, induction of labor. Values are means with ranges in brackets*.

### Materials

Primary antibodies against AKR1B1 and SLCO2A1 were produced and characterized in house (Kang et al., 2005). PTGS2 antibodies were purchased from Santa Cruz Biotechnology (2145 Delaware Avenue Santa Cruz, CA, 95060 USA). RhoGDI was used as a reference given its strong expression in human intrauterine tissues (Lartey et al., 2006). Antibodies' species of origin, source, catalog number and dilutions for immunochemistry and immunoblotting are summarized in Table 2.

**Table 2:** **Source and dilutions of primary antibodies used in immunohistochemistry (IHC) and immunoblotting (IB)**.

**Primary antibody**	**Species**	**Source (Catalog umber)**	**Dilution (IHC)**	**Dilution (IB)**
AKR1B1	Rabbit	Gift from Dr. Fortier	1:100	1:2000
hPGT	Rabbit	Gift from Dr. Fortier	1:50	1:1000
PGDH	Rabbit	Gift from Dr. Fortier	1:100	1:100
PGES	Rabbit	Cayman (160140)	1:50	1:1000
COX-2	Goat	Santa Cruz (sc-1745)	1:50	1:250
RhoGDI	Goat	Santa Cruz (sc-360)	_	1:20000

The secondary antibodies used were horseradish peroxidase conjugated (DAKO, Ely, UK). The rabbit anti-goat was used at 1:1000 dilutions, swine anti-rabbit was used at 1: 1000 and the rabbit anti mouse at 1:200. Cytokines (IL-1 and TNF-α, BioSource Europe, Belgium) were added to membranes in the explants to test their effects on PG production.

### Immunohistochemistry

Placental tissues were fixed with formalin for 24 h. Representative blocks of each tissue were taken and processed in the Histopathology Department at the Bristol Royal Infirmary with a Leica JUNG TP 1050 Tissue processor (Leica, Heidelberg, Germany) into paraffin wax and sectioned at 2 μm thickness.

The sections were dewaxed in Histo-Clear and passed through alcohol. Endogenous peroxidase activity was blocked by 1% hydrogen peroxide and then washed in running water. The antigen was retrieved by boiling in a microwave after immersion in 500 ml of citrate buffer solution. After washing with PBS 0.02% Tween, the slides were blocked in a solution containing 1% bovine serum albumin (BSA) plus 1% normal human serum and left at room temperature for 1 h.

The primary antibodies were prepared in PBS solution, added directly onto the sections and incubated overnight in a humid chamber at 4°C, then washed in PBS 0.02% Tween-20 three times each for 5 min. Secondary antibodies were added at room temperature for at least 1 h, and then washed in the same way as the primary antibodies. The slides were incubated with DAB solution for 10 min after which were dipped in water and then counter-stained with Mayer's hematoxylin for 30 s and washed with tap water. The slides were dehydrated in graded alcohol solutions and fixed in Histo-Clear, then mounted using mounting media and covered with coverslips. The primary antibodies were excluded during staining of the slides used as a negative control for the secondary antibodies.

Images were obtained by using a Leica DC300 camera (Leica, Heidelberg Germany) mounted on an Olympus BX40 microscope (Melville, NY, USA).

### Immunoblotting

The tissues were homogenized on ice with cold RIPA buffer (50 mM Tris pH 7.5, 150 mM NaCl, 1 mM EDTA, 1% NP40, 0.5% Triton × 100 and 1% SDS) then centrifuged for 40 min at 16,000×g and 4°C. The supernatant was collected and the protein concentration was determined by the bicinchoninic acid protein assay kit (Thermo Scientific, Rockford, IL, USA).

Homogenized samples (50 μg protein) were separated by SDS-PAGE (12% gel) and transferred onto Immobilon membranes (VWR international, Poole, UK). Membranes were blocked with WesternBreeze (Invitrogen) blocking buffer for 1 h at room temperature, and then incubated with diluted primary antibodies overnight at 4°C. After washing, the membranes were incubated with horseradish peroxidase-conjugated secondary antibodies (swine anti-rabbit, was used at 1: 5000 dilution and rabbit antigoat at 1:20000) for 2 h at room temperature. Membranes were then washed (three times for 5 min) with washing buffer and prepared for detection by chemiluminescence (Immobilon Western Chemiluminescent HRP Substrate, Millipore, Billerica, MA, US). PTGS2 protein was recognized by its antibody at 72 kDa whereas AKR1B1 protein was recognized by the antibody at 37 kDa and SLCO2A1 protein at 72 kDa. All detected bands were normalized to their individual RhoGDI bands, and then normalized to the ratio of PTGS2 to RhoGDI of a pooled sample used in all gels to eliminate the variations among different gels.

### Fetal membrane explants

Membranes were manually cut into discs (18 mm in diameter) and were held using silicone rubber rings in the upper chamber of a Transwell system (Costar) in which the original disc had been removed. In this model the choriodecidua faces the upper chamber, and the amnion faces the lower chamber. The mounted explant was then placed in a 12-well tissue-culture plate (Costar) (Zaga-Clavellina et al., 2007).

One milliliter of sterile Dulbecco's modified Eagle's medium (DMEM) supplemented with 10% fetal calf serum and antibiotic-antimycotic solution (penicillin, 100 U/ml; streptomycin, 100 μg/ml; amphotericin B, 0.25 μg/ml) was added to each chamber. The plates were incubated under 5% CO_2_ in 95% air at 37°C for 24 h to stabilize the tissues after manipulation.

### Cytokine exposure in explants and measurement of PGE_2_ and PGF_2α_

Pro-inflammatory cytokines stimulate prostaglandin synthase expression and PG release in human intrauterine tissues and we wished to examine the effect of cytokine exposure on both the maternal and fetal side of the fetal membranes on the rate of PGE_2_ and PGF_2α_ production (Norwitz et al., 1992; Phillips et al., 2011). The medium was changed to serum free DMEM, then explants were exposed to 10 ng/ml of IL-1β, or TNF. We tested different doses and we found that 10 ng/ml of IL-1β, or TNF were the appropriate doses that gave maximum stimulation without toxic effect on tissues. Each experiment included the following four conditions in duplicate for each membrane: (1) control membranes in which only medium was added to both compartments, (2) IL-1β, or TNF added to upper compartment (choriodecidua) only, (3) IL-1β, or TNF added to lower compartment (amnion) only, (4) IL-1β, or TNF added to both upper and lower compartments simultaneously. The media on both sides were collected after 6 h of stimulation and frozen at −80°C for prostaglandin assays. PGE_2_ and PGF_2α_ were measured in collected media using the ELISA technique. Incubation of the tissues for longer than 6 h did not show a further significant increase in prostaglandin production.

### Enzyme linked immunosorbent assay (ELISA) of prostaglandins

Measurement of PGE_2_ and PGF_2α_ was performed using an ELISA technique in which acetylcholinesterase-linked PG tracers were used as described previously (Asselin et al., 1996). Rabbit anti-PGE_2_ (kindly provided by Dr. T. G. Kennedy, University of Western Ontario, Ontario, ON, Canada) and sheep anti-PGF_2α_ (BioQuant, Ann Arbor, MI, USA) were used as selective antibodies. The inter- and intra-assay coefficients of variation (*n* = 12) were 16 and 10%, respectively. Briefly, 50 μl of collected culture medium was placed in a 96-well plate coated with goat anti-rabbit (PGE_2_) or rabbit anti-sheep (PGF_2α_) secondary antibody. A volume of 50 μl from each the tracer and the respective primary antibody was added to each sample after which the samples were incubated overnight at room temperature. After washing each well, 200 μl of Ellman's reagent (69 mM acetylthiocholine iodide; Sigma A5751, 5 g) and 54 mM 5,5'-dithiobis (2-nitrobenzoic acid; Sigma D8130, 5 g) dissolved in 10 mM phosphate buffer pH 7.4 was added. The plate was incubated on a shaker in the dark at room temperature, the reactions between the bound enzyme tracer and the Ellman's reagent yield a yellow color that can be measured with a photometric plate reader. A standard curve was used ranging from 39 to 5000 pg/ml PG.

### Statistical methods

The data was analyzed with SAS statistical software version 9.1.3. (SAS Institute Inc., Cary, NC, USA). The groups were analyzed separately, using Two-Way ANOVAs for each group. Pairwise comparisons were made between the control mean and each of the 9 treatment means; multiple comparisons were calculated using Dunnett's procedure.

## Results

### Localization of PGF synthase and PG transporter in intrauterine tissues at term

**Figure 1:**
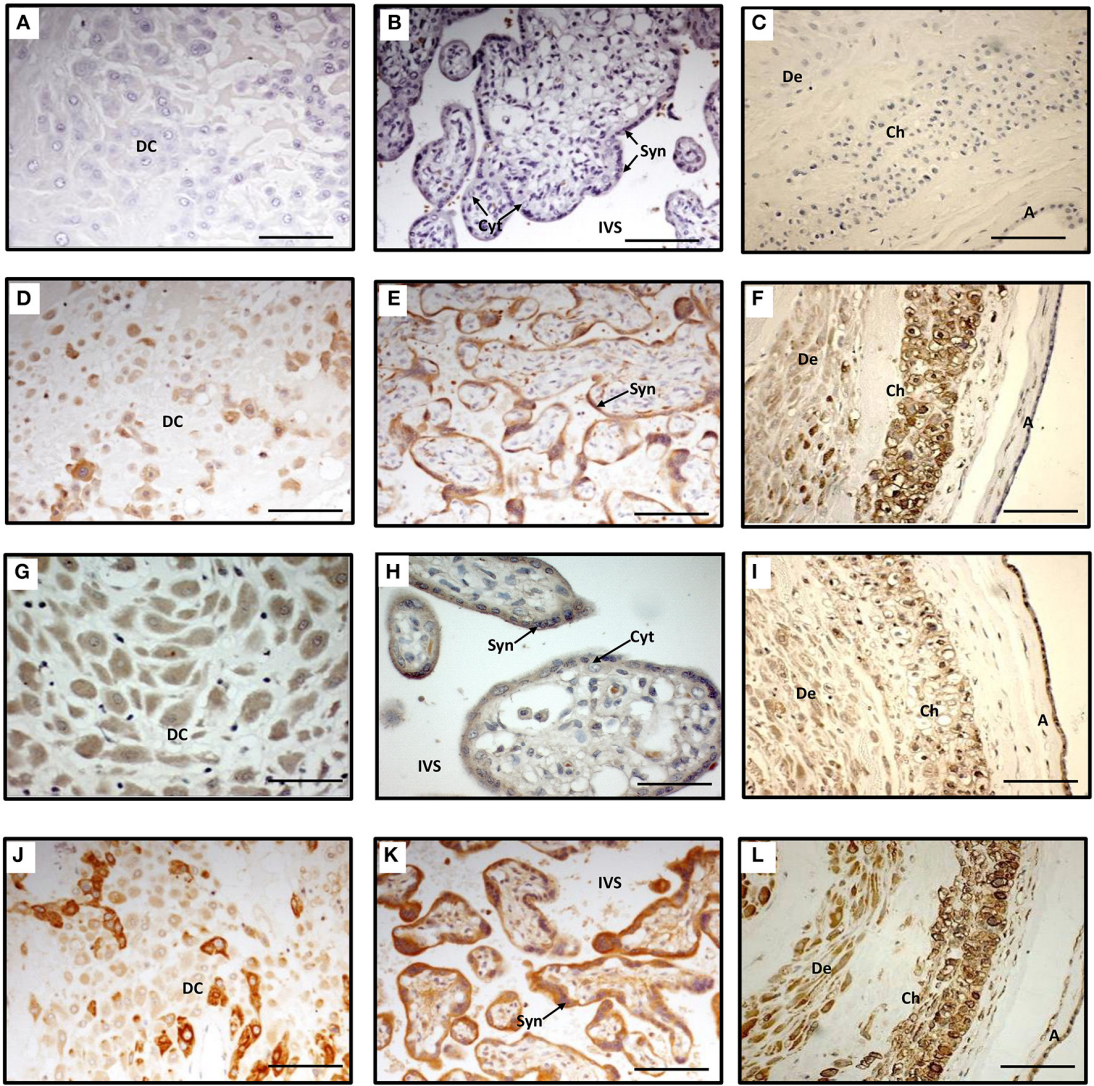
**Immunohistochemistry staining of term decidua basalis (A), chorionic villi (B) and fetal membranes (C) with AKR1B1 antibody (D–F), SLCO2A1 antibody (G–I), and PTGS2 antibody (J–L)**. Primary antibodies were omitted in **(A–C)** which served as controls. DC, decidual cells; Syn, syncytiotrophoblast; Cyt, cytotrophoblast; IVS, intervillous space; De, decidua parietalis; Ch, chorion; and A, amnion. Bar = 100 μm.

AKR1B1 and SLCO2A1 proteins were clearly expressed in decidual cells (Figures 1A–L). In decidual cytoplasm there was uneven distribution of AKR1B1 protein (Figure 1A) while the surrounding connective tissue appeared negative (Figure 1D). AKR1B1 protein was identified in the chorionic villi, in which the staining was strong in the cytoplasm of syncytiotrophoblasts while cytotrophoblasts and villous stroma were negative (Figure 1E). The SLCO2A1 protein was localized in decidual cytoplasm with no staining in the connective tissue (Figure 1G). The syncytiotrophoblasts in the chorionic villi showed weak staining in their cytoplasm and the cytotrophoblasts did not show any staining with SLCO2A1 antibodies (Figure 1H).

Staining with PTGS2 antibody was found to be strongly positive in the cytoplasm of some large decidual cells while smaller cells showed weak staining; connective tissue of decidua basalis showed no staining (Figure 1J). In the villi PTGS2 protein was localized in the cytoplasm, in the outer layer (syncytiotrophoblast) staining was intense, while the inner layer of the villi (cytotrophoblast) showed weak staining. Less intense staining was observed in the core of the villi (Figure 1K).

In the fetal membranes AKR1B1 was highly expressed in the chorion compared to decidua and there was no expression of this protein in the amnion while a poor staining was detected in the mesenchymal layer of the amnion (Figure 1F). The intensity of the staining with SLCO2A1 antibody was equal in the two layers of the fetal membranes and decidua parietals (Figure 1I).

All layers of the membranes and decidua parietals stained strongly positive with PTGS2 antibody, the staining was also observed in the mesenchymal layer of the amnion (Figure 1L).

### Effect of labor on the expression of PGF synthase and PG transporter in the fetal membranes

**Figure 2:**
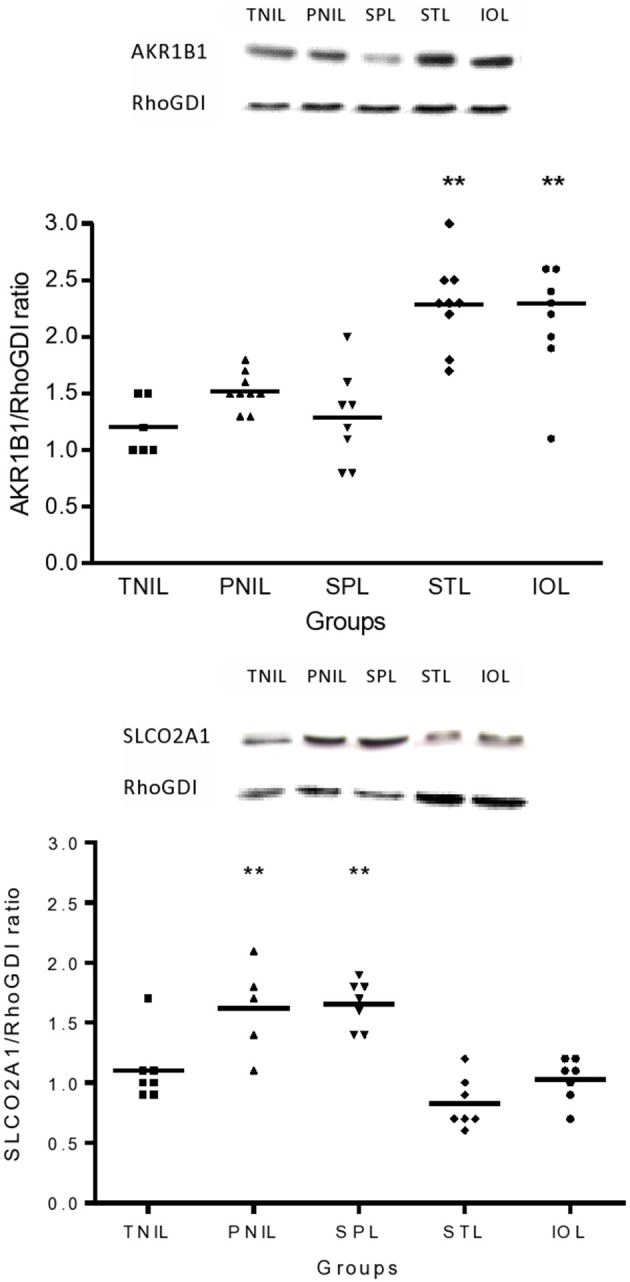
**Expression of AKR1B1 and SLCO2A1 protein in the fetal membranes in association with term and preterm labor compared to not in labor**. TNIL, term not in labor; PNIL, preterm not in labor; SPL, spontaneous preterm labor; STL, spontaneous term labor; IOL, induction of labor. Westerns are representative of immunoblots prepared from fetal membranes collected immediately after delivery, ^**^p < 0.005.

Using immunoblotting we found an increase in the expression of AKR1B1 protein in fetal membranes collected after term labor whether spontaneous or induced. However, preterm labor was not associated with an increase in the level of AKR1B1 protein (Figure 2). By contrast, SLCO2A1 protein was significantly decreased with advancing gestation with no changes in association with labor at term or preterm (Figure 2). The increase in AKR1B1 in the STL group and the apparent decrease in SPL reflected similar changes in PTGS2 protein expression in the same samples (data not shown).

### Effect of cytokines on PG production in the fetal membranes

**Figure 3:**
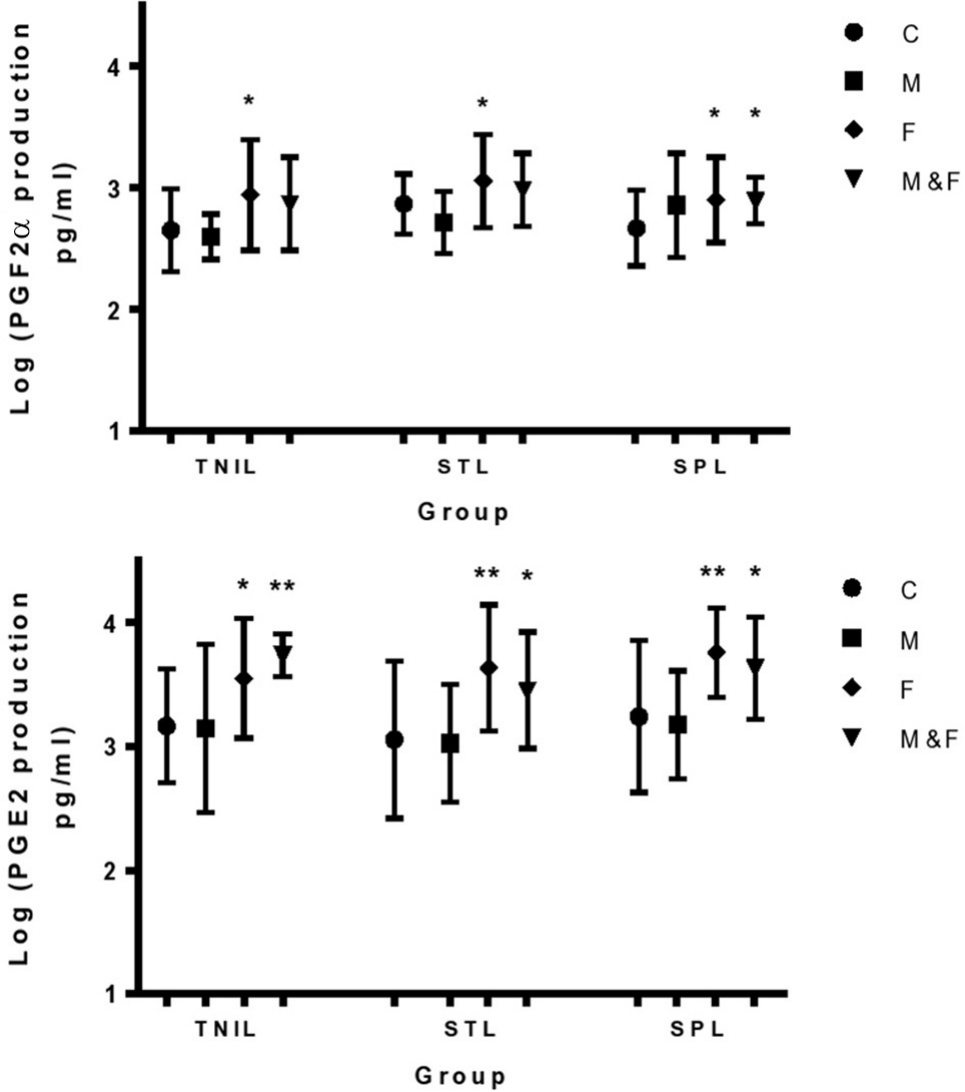
**Effect of IL-1β on PGE_2_ and PGF_2α_ production from the fetal side of membranes**. C, control; M, maternal side exposure; F, fetal side exposure; and M&F, exposure to maternal and fetal sides; TNIL, term not in labor; STL, spontaneous term labor; SPL, spontaneous preterm labor. The bars represent the mean ± SD; *n* = 4, ^*^p < 0.05 and ^**^p < 0.005.

**Figure 4:**
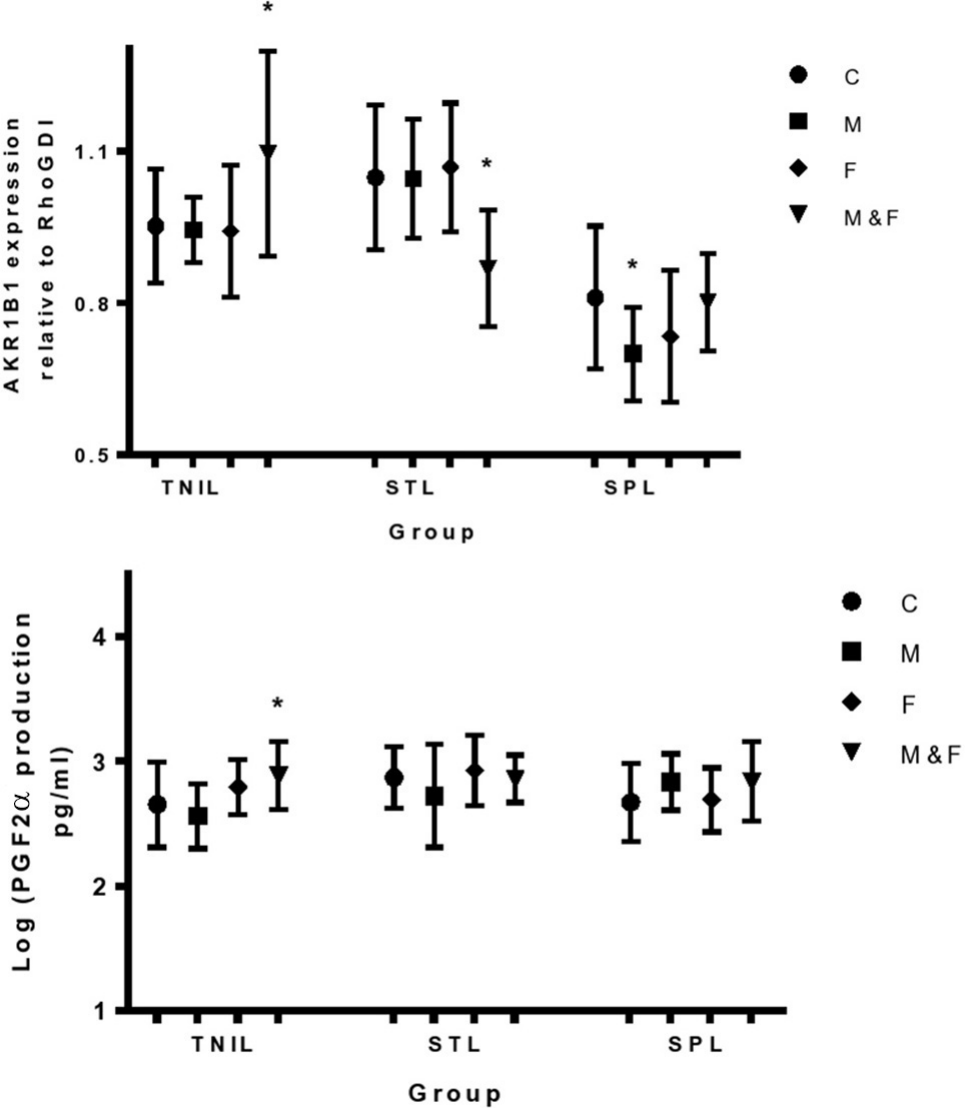
**Effect of TNF-α on expression of AKR1B1 protein in the fetal membranes and the associated changes in PGF_2α_ production from fetal side of membranes**. C, control; M, maternal side exposure; F, fetal side exposure; and M&F, maternal and fetal sides exposure. TNIL, term not in labor; STL, spontaneous term labor; SPL, spontaneous preterm labor. The bars represent the mean ± SD; *n* = 4, ^*^p < 0.05.

Exposure of the fetal side to IL-1β in all our tissues significantly increased PGF_2α_ and PGE_2_ production from the fetal side (Figure 3) and this was associated with a significant increase in PTGS2 protein expression. At term, before the onset of labor, TNF had a significant stimulatory effect on PGF_2α_ production from the fetal side when added to both sides of the membrane at the same time. This effect was associated with a significant increase in AKR1B1 expression in the TNIL group, but not in the STL and SPL groups (Figure 4).

## Discussion

To our knowledge this is the first study to compare the level of protein expression of the PGF synthase AKR1B1 and the PG transporter SLCO2A1 in fetal membranes of non-laboring and laboring women at term and preterm. We have found that expression of PTGS2 and AKR1B1 is increased in fetal membranes at term labor, whereas SLCO2A1 decreases with advancing gestation and term labor. Prior to labor, IL-1 enhances the expression of PTGS2; while in labor TNF stimulates the expression of PTGS2 and AKR1B1. During term labor AKR1B1 is upregulated with a concurrent downregulation of the prostaglandin transporter SLCO2A1. IL-1 and TNF have a stronger stimulatory effect on PG release in the amnion than in choriodecidua.

The fetal membranes are in close contact with the uterine decidua in an area estimated to be around 2000 cm^2^ and they are thought to have a central role in the process of human labor through paracrine effects on uterine activation (Myatt and Sun, 2010; Prince et al., 2014). It has been suggested that fetal membranes play a part in maintaining uterine quiescence by releasing putative inhibitors of spontaneous and oxytocin-induced uterine contractions (Carvajal et al., 2006). Any disturbance of membrane integrity by amniotomy or membrane sweeping is known to be associated with a rapid increase in prostaglandin production followed by initiation of labor (Mitchell et al., 1977). Recently it has been proposed that the fetal membranes are involved in a positive feedback loop that stimulates the release of biologically active glucocorticoids and PGs which eventually lead to maturation of fetal organs and initiation of labor (Myatt and Sun, 2010).

In this paper we have described the localization of the proteins involved in PGF_2α_ synthesis and transport in intrauterine tissues. Our findings show that in fetal membranes PTGS2 protein is highly expressed in the amnion, chorion and adjacent decidua while AKR1B1 expression is highest in the chorion and is absent in the amnion. The co-localization of PTGS2 and AKR1B1 in the chorion and adjacent decidua indicate that PGF_2α_ production at this site is important for initiation of human labor. In addition we have demonstrated that SLCO2A1 protein is expressed in the amnion and chorion as well as the decidua. Our observations are in line with a recent study which confirmed that human fetal membranes possess autoregulatory mechanisms for PGF_2α_ action (Breuiller-Fouche et al., 2010). The same study reported that in both layers of the fetal membranes the expression of AKR1B1 was higher than that of another PGF synthase, namely AKR1C3 (Breuiller-Fouche et al., 2010).

The current study shows increased density of AKR1B1 protein in fetal membranes with term labor and a decrease of SLCO2A1 protein with advancing gestation. SLCO2A1 contributes to the uptake of PG by the cells for their degradation and clearance (Kang et al., 2006) and its downregulation can lead to accumulation of PGs at their site of action. The downregulation of SLCO2A1 expression combined with upregulation of AKR1B1 protein in fetal membranes could have a synergetic effect to increase the levels of local PGF_2α_ during term labor. Compared to the recent findings of low expression of AKR1B1 in human myometrium (Phillips et al., 2011) our study demonstrates that AKR1B1 is highly expressed in the fetal membranes especially in association with term labor. Recently it has been suggested that AKR1B1 may act as a multifunctional enzyme in the fetal membranes through mediating inactivation of progesterone besides generation of PGF_2α_ (Breuiller-Fouche et al., 2010).

Our findings have shown an increase in AKR1B1 in fetal membranes after IOL which suggests that this change is part of the final steps in the process of labor at term. However, preterm labor occurs with low levels of AKR1B1; this may reflect differences in gestational maturity of the fetal membranes and increased sensitivity of the myometrium to potential triggers of labor such as PGF2α. We have also observed that PTGS2 protein expression was significantly increased in fetal membranes in association with term but not preterm labor, which supports the assumption that the mechanism of spontaneous labor is at least partially different in term and preterm groups.

Late pregnancy is a complex equilibrium between physiological pathways that maintain uterine quiescence and potential disruption by inflammatory pathways probably triggered by infection although many questions remain (Prince et al., 2014). IL-1 and TNF are key mediators of inflammation and immune responses and their potential role in labor has been emphasized earlier (Norwitz et al., 1992; Hansen et al., 1999). We have confirmed that IL-1β stimulates PGF2α and PGE2 production from fetal membranes before and after the onset of labor; this implies that IL-1β is an important factor for initiation as well as establishment of human parturition. A recent study reported that the increase in production of PGF2α and PGE2 after stimulation of human endometrial cells with IL-1β can be reduced in the presence of FP receptor antagonists, indicating that PGF2α was able to stimulate its own and PGE2 productions (Bresson et al., 2012). Other workers found that IL-1β was a potent stimulator of PGE2 production from term intact fetal membranes collected before the onset of labor (Brown et al., 1998). Previous studies reported that IL-1β increased in the amniotic fluid of spontaneous term (Romero et al., 1992) as well as preterm labor (Baud et al., 1999). Another group of investigators reported that IL-1β increased oxytocin release from human decidua by a mechanism that involved production of PGs through PTGS2 upregulation (Friebe-Hoffmann et al., 2007). This local interaction between PGs and oxytocin may facilitate the progress of labor.

We have observed a moderate effect of TNF to initiate term labor through upregulation of AKR1B1 and stimulation of PGF2α production. In line with our results, Sadowsky and colleagues, using intra-amniotic infusions of pro-inflammatory cytokines demonstrated that IL-1β was more effective than TNF in initiating preterm labor in rhesus monkeys (Sadowsky et al., 2006). Surprisingly, we observed that TNF inhibits AKR1B1 protein expression in membranes collected after labor when added to both sides in term and maternal side in preterm. This inhibition might be an important mechanism to control the level of PGF2α during labor since high levels of PGF2α might be harmful to the fetus if it triggered tetanic contractions.

Interestingly, in the current study we noticed that although the exposure of the fetal side of term fetal membranes to IL-1β stimulated PGF2α production, the simultaneous exposure of fetal and maternal sides to IL-1β had no effect. Osman and coworkers reported that although the expression of IL-1β mRNA did not change in the choriodecidua (maternal side), its level was found to be significantly higher in the amnion (fetal side) after the onset of term labor (Osman et al., 2003). By contrast, the exposure of preterm membranes to IL-1β from both sides had an accumulating stimulatory effect for PGF2α production providing further evidence for different mechanisms for term and preterm labor.

We observed that during term and preterm labor the amnion became more responsive to the stimulatory effect of IL-1β and released high levels of PGE2 when compared to not in labor membranes. This observation might indicate that the process of term and preterm labor involves upregulation of IL-1β receptors in the amnion.

Our findings suggest that the sequence of IL-1β effects in the fetal membranes is to stimulate PTGS2 expression by autocrine or paracrine action in choriodecidua, followed by paracrine stimulation of PG release in the amnion. In this regard Zaga and coworkers reported that IL-1β secretion to the amniotic compartment was observed only when choriodecidua and amnion were together. On the other hand when both tissues were mechanically separated, only choriodecidua secreted IL-1β (Zaga et al., 2004).

A previous study demonstrated that PTGS2 mRNA progressively accumulates in the membranes before and during labor causing a steady rise in PG biosynthetic capacity when translated into active protein (Johnson et al., 2002). Our findings imply that IL-1β released by fetal tissues, might be one of the factors that can stimulate PTGS2 expression via its action on the amnion. On the other hand, concurrent exposure of maternal and fetal sides of the membrane to TNF upregulated AKR1B1 protein in the membranes of non-laboring women, which explains the stimulatory effect of this cytokine on PGF_2α_ production.

The data in the current study support the hypothesis that fetal membranes, through their response to stimulation by pro-inflammatory cytokines and release of PGs, play an important role in the process of human labor. Pro-inflammatory cytokines released by fetal tissues could be important triggering factors that need to receive focused attention in future research in an attempt to find more effective targets for the management of preterm labor.

In conclusion, advancing gestation is associated with accumulation of PTGS2 protein as well as a decrease of SLCO2A1 protein that helps to prepare the fetal membranes to respond to stimulatory factor(s) in labor. TNF acts during labor to increase AKR1B1 protein level leading to increased PGF_2α_ production. We can speculate that the trigger of labor is probably coming from the fetal side of the membranes when IL-1β is released and leads to synthesis of PTGS2 protein which starts labor by increasing PG production. Increased PGE_2_ and PGF_2α_ production as reflected by rising amniotic fluid concentrations may promote membrane rupture and initiate uterine contractions.

Parturition must be the result of maturational events in the fetus, probably involving the hypothalamic pituitary adrenal axis, increased sensitivity of the uterus to stimulatory agonists and loss of inhibitory mechanisms in myometrial smooth muscle (Lopez Bernal et al., 1995; Li et al., 2014). In a minority of cases inflammatory infiltration of the fetal membranes and decidua may trigger premature labor, however many women go into preterm labor without signs of infection or inflammation and other mechanisms may be involved. In this regard it is interesting to note that oxytocin stimulates PTGS2 expression through a calcium dependent NFAT (nuclear factor of activated T cells) signaling pathway (Pont et al., 2012). Our findings also indicate that the mechanisms of spontaneous labor are different at term and preterm. Further research into the paracrine and autocrine effects of cytokines and PGs in the decidua/fetal membranes area is required to elucidate the biochemical and physiological events underlying these mechanisms.

## Author contibutions

Hana A. Alzamil and A. López Bernal designed the protocols. H. Alzamil conducted the studies, data analyses and drafted the manuscript. A. López Bernal interpreted the data, revised and modified the draft critically. Joya Pawade interpreted the immunohistochemical data. Michel A. Fortier revised the draft critically. All authors approved the version to be published.

## Funding

Hana A. Alzamil was supported by a Fellowship from King Saud University and the results were incorporated into a PhD thesis at the University of Bristol. The work was supported by Wellbeing of Women (grant RG825).

### Conflict of interest statement

Michel Fortier has a patent for methods for the regulation of the prostaglandin F synthase (PGFS) activity of AKR1B1 and uses thereof. The authors declare that the research was conducted in the absence of any commercial or financial relationships that could be construed as a potential conflict of interest.
